# Effects of maternal-offspring supplementation of probiotics and synbiotics on the immunity of offspring Bama mini-pigs

**DOI:** 10.3389/fimmu.2025.1507080

**Published:** 2025-02-13

**Authors:** Sujuan Ding, Ting Ye, Md. Abul Kalam Azad, Qian Zhu, Yating Liu, Bie Tan, Xiangfeng Kong

**Affiliations:** ^1^ Key Laboratory of Agro-Ecological Processes in Subtropical Region, Hunan Provincial Key Laboratory of Animal Nutritional Physiology and Metabolic Process, Institute of Subtropical Agriculture, Chinese Academy of Sciences, Changsha, China; ^2^ College of Animal Science and Technology, Hunan Agricultural University, Changsha, China; ^3^ College of Advanced Agricultural Sciences, University of Chinese Academy of Sciences, Beijing, China

**Keywords:** Bama mini-pigs, immunity, maternal-offspring, probiotics, synbiotics

## Abstract

Maternal nutrition is one of the main factors regulating the growth and immunity of piglets. This study aimed to investigate the effects of maternal or maternal-offspring supplementation of antibiotics, probiotics, and synbiotics on the immunity of offspring (21, 65, and 125 day-old) in Bama mini-pigs. The results showed that adding antibiotics to maternal diets increased the plasma IFN-γ level of offspring piglets at 21 day-old. Compared with maternal supplementation, maternal-offspring supplementation of antibiotics decreased the IL-10 level in the spleen, probiotics decreased IL-2, IL-10, and TNF-α levels in the ileum, and synbiotics decreased IL-10 and IFN-γ levels in the ileum of offspring piglets. Moreover, maternal-offspring antibiotics supplementation increased the IL-1β level in the ileum, while probiotics supplementation increased the IL-1β level in the spleen of offspring piglets. Maternal antibiotics supplementation increased the TNF-α level in the ileum at 95 day-old compared with maternal probiotics and synbiotics supplementation. Maternal-offspring antibiotics supplementation increased the IL-1β level in the ileum compared with the probiotics supplementation, while synbiotics supplementation increased the IL-6 level in the ileum than the probiotics and antibiotics supplementation at 95 day-old. Moreover, maternal-offspring probiotics supplementation increased the IL-1β level in the spleen of offspring pigs, which was higher than the maternal probiotics supplementation. These findings suggest that the immune function of the offspring piglets varied depending on the specific approach used for probiotics and synbiotics supplementation.

## Introduction

The immature immune system and different common stressors at birth and weaning transition, including physiological, nutritional, and environmental factors, increase the risk of infection of piglets, which can seriously lead to intestinal and respiratory diseases, and even death (1). The nutritional and physiological status of sows during pregnancy and lactation is directly associated with the development and disease resistance of fetuses and neonates (2). During pregnancy and lactation, maternal nutrient intake shapes the development of the fetal immune system (3). In mammals, almost all nutrients, respiratory gases, excretory products, and exogenous drugs are transported through the umbilical cord during the development of the fetus (4). After birth, the foremost function of suckling is to support the essential nutrients of newborns. Piglets are born without accumulated brown fat, while breast milk can provide energy promptly (2). At the same time, colostrum also provides bioactive molecules such as immunoglobulins for piglets (5). Although maternal immunoglobulins cannot be transported through the placental barrier (6), these immunoglobulins are transferred to piglets through colostrum (7). In addition, maternal diets influence the maturation of the neonatal immune system by regulating the composition of colostrum and milk (8). Therefore, maternal nutrition is crucial for developing the immune function of offspring.

Probiotics are defined as “a living microbe that, when added in an adequate amount, is beneficial to the health of the host” and are considered an economically safe alternative to antibiotics to improve the host’s health (9). It is well-established that probiotics regulate the host’s immune system by stimulating immune functions and inhibiting pathogens. Research evidence indicated that probiotics can protect the host from different infectious and non-communicable diseases through their colonization and inhibition of pathogens (10). For instance, *Lactobacillus salivarius* has been found to improve immunomodulatory function by enhancing survival in the ileum through the competitive advantage conferred by its bacteriocins (11). Synbiotics, as a mixture of probiotics and prebiotics, enhance the survival and colonization of active microbial components in the gut by selectively stimulating growth and/or activating the metabolism of one or a limited number of health-promoting bacteria, thereby having beneficial effects on the host health (12). Currently, the primary purpose of using probiotics and synbiotics in the pig industry is to improve the overall health status of animals (13). However, when selecting dietary supplementation with probiotics, it is necessary to evaluate their potential beneficial effects and related metabolism. Moreover, determining synbiotics formulas is a complex and challenging task involving animal breed and growth stages.

China has rich resources of indigenous pig breeds that are highly adaptable to the environment, and their high-quality meat has attracted the interest of consumers. However, the current production management for indigenous pig breeds is not well-developed, which may be one of the most important reasons why the consumer acceptance of indigenous pig breeds is not widely established. The Bama mini-pig is a domestic pig breed in Bama County, Guangxi, China, which is a unique breed with long-term reproductive success (14). Bama mini-pigs have advantageous characteristics with red muscle, white fat, and delicious taste; however, the immune dysfunction and slow growth rate restrict the overall productivity of Bama mini-pigs (15). Thus, we hypothesized that maternal-offspring supplementation of probiotics and synbiotics might have positive regulatory effects on the immune function of offspring in Bama mini-pigs. Therefore, the present study was designed to evaluate the effects of maternal or maternal-offspring supplementation of probiotics and synbiotics on the immunity of offspring piglets.

## Materials and methods

### Animals and study design

Sixty-four Bama mini-pigs within their 3^rd^-5^th^ parity were randomly allocated into four groups, and each group consisted of 16 replicates. Sows were fed either a basal diet (CON group) or a basal diet supplemented with 50 g/t·feed virginiamycin (ANT group), 200 mL/d·head probiotic culture solution (PRO group), and 500 g/t·diet xylo-oligosaccharide and 200 mL/d·head probiotic culture solution (SYN group), respectively. The feed additives were added to sow diets throughout pregnancy (three days after mating) and lactation periods. The probiotic fermentation liquid was provided by Hunan Lifeng Bio-technology Co., Ltd. (Changsha, China), containing *Lactobacillus plantarum* ≥ 1×10^8^ CFU/mL and *Saccharomyces cerevisiae* ≥ 0.2×10^8^ CFU/mL. Xylo-oligosaccharides (XOS, including xylobiose, xylotriose, and xylotetrose; >35%) was provided by Shandong Longlive Bio-technology Co., Ltd. (Shandong, China).

According to the management standards of the pig farm, sows were fed at 8:00 and 17:00 daily, with free access to drinking water. The feeding amount for each sow during pregnancy was 0.8 kg/d for 1−15 days, 1.0 kg/d for 16−30 days, 1.2 kg/d for 31−75 days, 1.5 kg/d for 76−90 days, and 2.0 kg/d for 91−110 days kg/d. The sows were fed 1.0 kg/d for one week before parturition, *ad libitum* three days after parturition, and 2.4 kg/d from delivery to weaning. After weaning (28 day-old), two piglets (one male and one female) close to the average body weight of the litter were selected from each litter for subsequent feeding experiments. Offspring piglets originated from the same treatment; two piglets from each litter and a total of four piglets from two litters were merged into one pen; each pen consisted of four piglets, and each group had eight pens.

Offspring piglets from the same maternal treatment group were randomly allocated into two treatments, including maternal supplementation and maternal-offspring supplementation. The feed additives supplementation levels for offspring piglets were as follows: 30 mL/d·head probiotics for PRO group and 250 g/t·feed XOS + 30 mL/d·head probiotics mixture for SYN group during 35−95 day-old; 60 mL/d·head probiotics for PRO group and 250 g/t·feed XOS + 60 mL/d·head probiotics mixture for SYN group during 96−125 day-old; 40 g/t·feed virginiamycin for ANT group during 35−125 day-old.

The composition and nutrient levels of the basal diet for sows and offspring piglets are presented in **Tables 1** and **2**. All pigs were raised in the same environment with the same feeding and management conditions. Breeding and management were carried out according to the standards of commercial pig farms.

**Table 1:** Composition and nutrient levels of basal diets for sows during pregnancy and lactation (air-dried; %).

Items	Pregnancy diet	Lactating diet
Ingredients, %		
Corn	37.50	66.00
Soybean meal	9.50	25.00
Wheat bran	14.00	5.00
Barley	25.00	0.00
Soybean hull	10.00	0.00
Pregnant sows’ premix^1^	4.00	0.00
Lactating sows’ premix^2^	0.00	4.00
Total	100.00	100.00
Nutrient levels^3^		
Digestible energy, MJ/Kg	12.55	13.87
Crude protein	12.82	16.30
Crude fiber	4.56	2.87
SID Lysine	0.48	0.75
SID Methionine + Cysteine	0.43	0.51
SID Threonine	0.37	0.53
SID Tryptophan	0.13	0.17
Calcium	0.62	0.65
Phosphorus	0.47	0.50

^1^Premix provided the following per kilogram of pregnant sow diet: CaHPO_4_·2H_2_O, 10 g; NaCl, 4 g; CuSO_4_·5H_2_O, 80 mg; FeSO_4_·H_2_O, 360 mg; ZnSO_4_·H_2_O, 240 mg; MnSO_4_·H_2_O, 100 mg; MgSO_4_·7H_2_O, 1 g; 1% ICl, 50 mg; 1% Na_2_SeO_3_, 36 mg; 1% CoCl_2_, 16 mg; NaHCO_3_, 1.4 g; vitamin A, 10 000 IU; vitamin D_3_, 1 800 IU; vitamin E, 20 mg; vitamin K_3_, 2.4 mg; vitamin B_1_, 1.6 mg; vitamin B_2_, 6 mg; vitamin B_6_, 1.6 mg; vitamin B_12_, 0.024 mg; folic acid, 1.2 mg; nicotinamide, 20 mg; pantothenic acid, 12 mg; biotin, 0.12 mg; ferrous glycinate, 100 mg; choline chloride, 1 g; phytase, 200 mg; flavor, 80 mg; and limestone, 12 g.

^2^Except for not containing 1 g MgSO_4_·7H_2_O and containing 1.5 g lysine for each kilogram of diet, the premix for lactating sow diet is the same as that for pregnant sows.

^3^Nutrient levels calculated using the NRC (2012) feed composition values; SID, standardized ileal digestibility.

**Table 2:** Composition and nutrient levels of the basal diet for weaned piglets (air dried; %).

Items	35−95 day-old	96−125 day-old
Ingredients		
Corn	54.92	58.00
Soybean meal	22.00	18.35
Wheat bran	10.13	11.35
Rice bran	8.95	8.30
Premix^1^	4.00	4.00
Total	100.00	100.00
Nutrient levels^2^		
Digest energy, MJ/kg	13.50	13.42
Crude protein	16.13	14.90
SID Lysine	1.40	1.30
SID Methionine + Cysteine	0.69	0.66
SID Threonine	0.78	0.74
Calcium	0.45	0.44
Total phosphorus	0.49	0.49

^1^Premix provided the following per kilogram of diets: enzymic preparation 1.2 g; vitamin A, 26 000 IU; vitamin D_3_, 10 000 IU; vitamin E, 70 IU; vitamin K_3_, 10 mg; vitamin B_1_, 10 mg; vitamin B_2_, 25 mg; vitamin B_6_ 10 mg; vitamin B_12_ 0.075 mg; biotin 0.4 mg; lysine, 7 g; methionine, 2 g; threonine, 3 g; tryptophan, 0.5 g; folic acid, 5 mg; nicotinamide, 100 mg; pantothenic, 50 mg; choline, 1 600 mg;flavouring agent, 500 mg; edulcorant, 300 mg; acidulating agent, 5 g; Cu (as CuSO_4_·5H_2_O), 230 mg; Mn (as MnSO_4_·H_2_O), 97 mg; Zn (as ZnSO_4_·H_2_O), 218 mg; Fe (as FeSO_4_·H_2_O), 165 mg; I (as Ca(IO_3_)_2_), 0.3 mg; Se (as Na_2_SeO_3_), 0.3 mg; Co (as CoSO_4_·xH_2_O), 0.4 mg; glucose, 2.1 g; antioxidants, 0.4 g; antimildew agent, 1 g; Ca (as CaHPO_4_ and CaCO_3_), 3.42 g; and P (as CaHPO_4_), 1.155 g. P (as CaHPO_4_) 1.155 g.

^2^Nutrient levels are calculated values.

### Sample collection

One piglet from each pen and eight piglets from each group at 21 day-old, 95 day-old (two months after weaning), and 125 day-old (three months after weaning) were selected for sampling. The body weight was measured after 12 h fasting. Blood samples (six piglets from each group) were collected into heparin anticoagulant tubes from the anterior vena cava, centrifuged at 4°C and 3500 × *g* for 10 min to obtain the plasma, and immediately stored at −80°C for immunocytokine assays. Experimental pigs were anesthetized by injection of Zoletil^@^ 50 (1 ml, 0.5 mg/kg; Beijing Lab Anim Tech Develop Co., Ltd., Beijing, China) at 21 day-old and the pigs at 95 and 125 day-old were euthanized using electrical stunning (120 V, 200 HZ) and exsanguination. After dissection, 0.5 g of spleen and liver tissues were collected from the same location, and the posterior end of the ileum (approximately 2 cm in length) was immediately removed and washed with phosphate buffer saline to remove the intestinal contents. Tissue samples were immediately preserved at −80°C for immunocytokine and gene expression analyses.

### Analysis of immunoglobulin and immunocytokine levels in plasma, spleen, and ileum

The immunoglobulin and immunocytokine levels in plasma, spleen, and ileum were determined with enzyme-linked immunosorbent assay (ELISA) kits. The testing protocols were followed as provided by the manufacturer’s instructions (Shanghai Kexing Trading Co., Ltd., Shanghai, China). The absorbance values (OD at 450 nm) were read on a microplate reader (Infinite M200 PRO, TECAN, Männedorf, Switzerland). The immunoglobulin and immunocytokine levels in plasma were expressed as the unit of volume. The total protein level in the spleen and ileum was measured using the BCA kit (Beyotime, Shanghai, China), and the final levels of immunoglobulins and immunocytokines in tissues were expressed as the unit of protein level.

### Analysis of immune-related gene expressions in the ileum

The real-time quantitative PCR (RT-PCR) was used to detect the expression of immune-related genes in the ileum following the methods described previously (16). The total RNA from the ileal tissues was extracted with Trizol reagent (Accurate Biology, Changsha, China), and the agarose gel electrophoresis was used to detect the quality of the extracted RNA. According to the manufacturer’s instructions (Accurate Biology, Changsha, China), the extracted RNA was reverse-transcribed at 37°C for 15 min and 95°C for 5 s. An RT-PCR was conducted using the SYBR^®^ Green Premix Pro Taq HS qPCR kit (Accurate Biology, Changsha, China) and LightCycle^®^ 480 II Real-Time PCR system (Roche, Basel, Switzerland). The primers used in this study are presented in **Supplementary Table S1**. The PCR cycle conditions for RT-PCR were as follows: initial denaturation at 94°C for 30 s, denaturation at 94°C for 5 s and annealing at 55°C for 30 s with 40 cycles, and a final extension for 30 s at 72°C. The relative expression levels of the target gene and β-actin were calculated by comparing Ct values, and expressed by 2^-ΔΔCt^ values (15).

### Statistical analysis

All data are expressed as means with their standard error of the means (SEM). The individual piglets were considered the experimental unit. Statistical analyses were performed by two-way ANOVA for pig breed and day-old using the SPSS 26.0 software package (SPSS, Inc., Chicago, IL, USA). *P*-values < 0.05 were considered statistical significance.

## Results

### Effects of maternal supplementation of probiotics and synbiotics on the immune response in the spleen and ileum of offspring piglets at 21 day-old

The effects of maternal supplementation of probiotics and synbiotics on immunocytokines and immunoglobulins levels in the spleen and ileum of piglets at 21 day-old are presented in **Tables 3** and **4**. In the spleen, interleukin (IL)-6 and interferon (IFN)-γ levels were higher (*P* < 0.05) in the SYN group compared with the other three groups, while IL-6 level in the PRO group and IFN-γ level in the ANT group were higher (*P* < 0.05) compared with the CON group. The SYN group had higher (*P* < 0.05) IL-2 and tumor necrosis factor (TNF)-α levels than the CON and ANT groups, whereas the TNF-α level was higher (*P* < 0.05) in the PRO group compared with the CON group. Moreover, the IL-10 level was lower (*P* < 0.05) in the CON group, whereas the IL-1β level was higher (*P* < 0.05) in the SYN group when compared with the other groups (**Table 3**). However, there were no significant differences (*P* > 0.05) in the immunocytokine (**Table 3**) and immunoglobulin (**Table 4**) levels in the ileum of piglets at 21 day-old among the four groups.

**Table 3:** Immunocytokines level in the spleen and ileum of offspring piglets at 21 day-old.

Items	CON	ANT	PRO	SYN	SEM	*P*-values
Spleen						
IL-1β (μg/mg)	6.10^B^	7.52^B^	7.46^B^	9.32^A^	0.55	0.005
IL-2 (pg/mg)	79.97^B^	86.98^B^	95.85^AB^	106.55^A^	5.71	0.021
IL-6 (μg/mg)	175.87^C^	221.24^BC^	241.97^B^	331.56^A^	15.56	<0.001
IL-10 (μg/mg)	23.23^B^	32.18^A^	31.51^A^	37.97^A^	2.18	0.001
IFN-γ (pg/mg)	460.66^C^	558.45^B^	542.95^BC^	747.13^A^	29.74	<0.001
TNF-α (pg/mg)	77.41^C^	87.84^BC^	101.40^AB^	110.11^A^	6.88	0.015
Ileum						
IL-1β (μg/mg)	12.23	12.90	12.35	9.92	1.18	0.317
IL-2 (pg/mg)	122.69	124.19	132.51	118.79	9.32	0.766
IL-6 (μg/mg)	314.60	364.49	399.51	282.50	35.61	0.128
IL-10 (μg/mg)	22.09	26.06	19.14	21.58	2.49	0.293
IFN-γ (pg/mg)	311.38	359.29	361.89	375.35	26.07	0.353
TNF-α (pg/mg)	57.27	67.65	65.78	66.99	4.68	0.386

Data are expressed as means with their pooled SEM (*n* = 6). Different letters (A−C) indicate significant differences among groups (*P* < 0.05). CON, control group; ANT, antibiotics group; PRO, probiotics group; SYN, synbiotics group; IL, interleukin; IFN, interferon; TNF, tumor necrosis factor.

**Table 4:** Immunoglobulins level in the ileum of offspring piglets at 21 day-old.

Items	CON	ANT	PRO	SYN	SEM	*P*-values
sIgA (μg/mg)	4.72	6.10	4.70	5.65	0.48	0.125
IgA (pg/mg)	5.58	6.30	4.97	5.21	0.66	0.518
IgG (μg/mg)	55.49	65.92	47.94	56.92	6.67	0.328
IgM (pg/mg)	6.73	8.50	6.42	6.94	0.80	0.287

Data are expressed as means with their pooled SEM (*n* = 6). CON, control group; ANT, antibiotics group; PRO, probiotics group; SYN, synbiotics group; Ig, immunoglobulin; sIgA, secretory immunoglobulin A.

The expression levels of immune-related genes in the spleen and ileum of piglets at 21 day-old are presented in **Table 5**. The *TNF-α* expression was upregulated (*P* < 0.05) in the spleen of the PRO and SYN groups compared with the CON and ANT groups, while toll-like receptors (*TLR*)*4* expression was upregulated (*P* < 0.05) in the spleen of the SYN groups than the other three groups. In the ileum, *IL-2* and myeloid differentiation factor 88 (*MyD88*) expressions were upregulated (*P* < 0.05) in the SYN group than in the other three groups. Moreover, the nuclear factor kappa B (*NF-κB*) expression was downregulated (*P* < 0.05) in the PRO and SYN groups than the CON group, whereas it was even more downregulated (*P* < 0.05) in the PRO group compared with the ANT group.

**Table 5:** Expression of immune-related genes in the spleen and ileum of offspring piglets at 21 day-old.

Items	CON	ANT	PRO	SYN	SEM	*p*-values
Spleen						
*IL-1β*	1.00	0.83	1.53	1.60	0.23	0.088
*IL*-2	1.00	0.94	1.35	1.49	0.21	0.221
*IL*-6	1.00	0.84	0.98	0.89	0.09	0.628
*IL*-10	1.00	0.97	1.30	1.29	0.17	0.129
*TNF-α*	1.00^C^	1.27^BC^	1.64^AB^	1.76^A^	0.16	0.003
*TLR*2	1.00	1.17	1.00	1.25	0.08	0.151
*TLR*4	1.00^B^	1.13^B^	1.11^B^	1.72^A^	0.12	0.001
*TRAF*6	1.00	0.93	1.06	0.89	0.07	0.057
*MyD*88	1.00	1.03	1.05	1.06	0.08	0.774
*NF-κB*	1.00	1.02	1.12	1.17	0.07	0.174
Ileum						
*IL-1β*	1.00	0.62	1.47	1.18	0.37	0.435
*IL*-2	1.00^B^	0.93^B^	1.13^B^	2.05^A^	0.27	0.022
*IL*-6	1.00	0.87	1.20	0.98	0.15	0.460
*IL*-10	1.00	1.16	1.11	0.96	0.18	0.843
*TNF-α*	1.00	0.86	1.20	0.83	0.13	0.417
*TLR*2	1.00	0.90	0.85	1.16	0.17	0.403
*TLR*4	1.00	0.83	0.71	0.73	0.17	0.585
*TRAF*6	1.00	0.84	0.69	1.17	0.18	0.273
*MyD*88	1.00^B^	0.98^B^	1.29^B^	2.10^A^	0.28	0.022
*NF-κB*	1.00^A^	0.75^AB^	0.41^C^	0.50^BC^	0.10	0.001

Data are expressed as means with their pooled SEM (*n* = 6). Different letters (A−C) indicate significant differences among groups (*P* < 0.05). CON, control group; ANT, antibiotics group; PRO, probiotics group; SYN, synbiotics group; IL, interleukin; TNF, tumor necrosis factor; TLR, toll-like receptors; TRAF6, tumor necrosis factor receptor-associated factor 6; MyD88, myeloid differentiation factor 88; NF-κB, nuclear factor kappa B.

### Effects of maternal-offspring supplementation of probiotics and synbiotics on plasma immunocytokines of offspring piglets at 95 and 125 day-old

**Figure 1:**
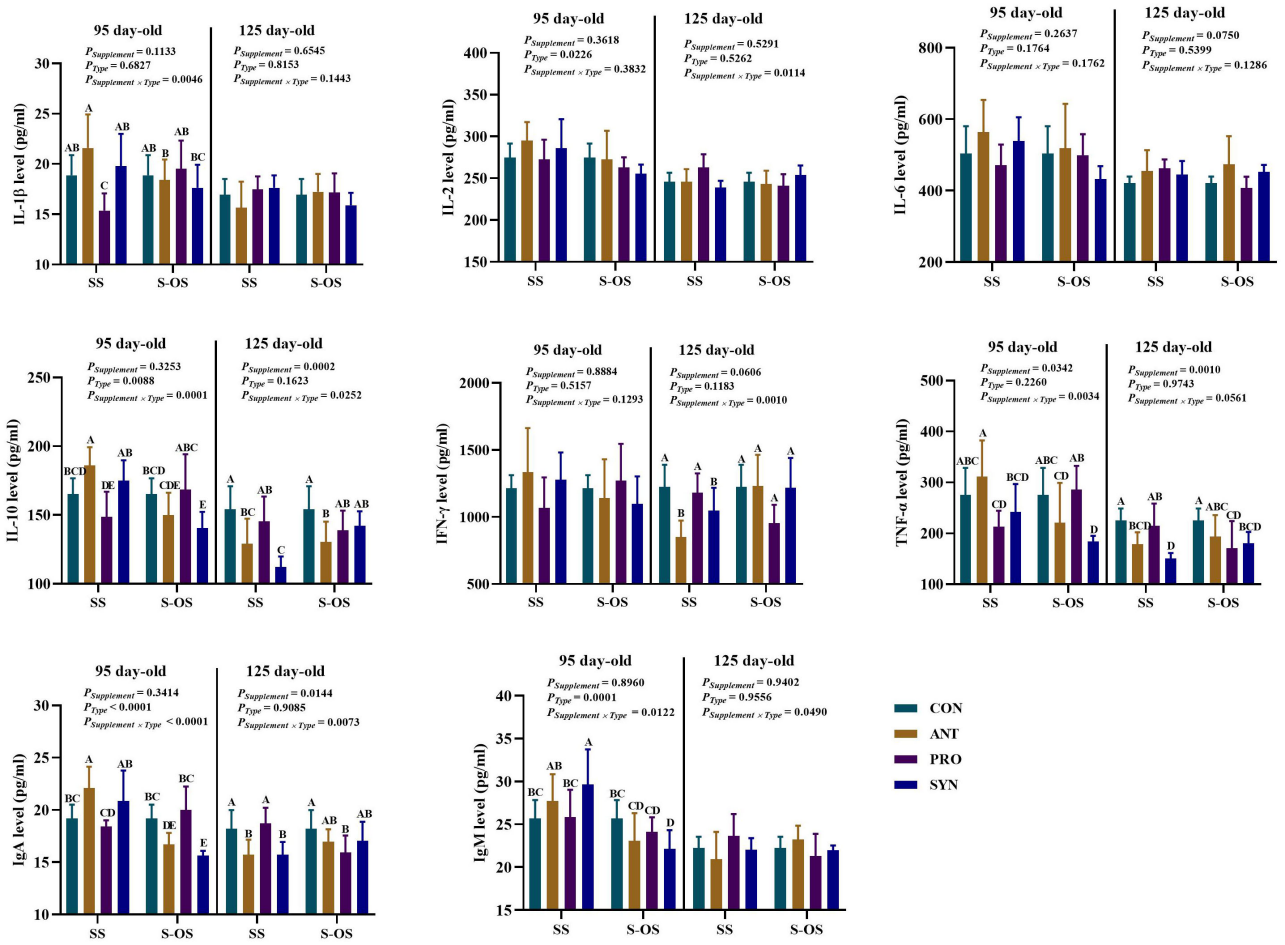
Effects of maternal-offspring supplementation of probiotics and synbiotics on plasma immunocytokines and immunoglobulins of offspring piglets at 95 and 125 day-old. Data are expressed as means with their pooled SEM (*n* = 6). Different letters (A−E) indicate significant differences among groups (*P* < 0.05). *P*_Supplement_: *P*-values of different kinds of additives in the diet, including no or added antibiotics, probiotics, or prebiotics; *P*_Type_: *P*-values of different ways of supplement, including maternal supplementation and maternal-offspring supplementation; *P*_Supplement_ × *P*_Type_: *P*-values of different kinds of additives and different ways of supplement. CON, control group; ANT, antibiotic group; PRO, probiotics group; SYN, synbiotics group; SS, maternal supplementation; S-OS, maternal-offspring supplementation; IL, interleukin; IFN, interferon; TNF, tumor necrosis factor; Ig, immunoglobulin; sIgA, secretory immunoglobulin A.

The effects of maternal-offspring supplementation of probiotics and synbiotics on plasma immunocytokines of offspring pigs at 95 and 125 day-old are shown in **Figure 1**. At 95 day-old, the maternal-offspring supplementation decreased (*P* < 0.05) the plasma IL-1β, IL-10, TNF-α, immunoglobulin (Ig)A, and IgM levels in the ANT group, as well as the plasma IL-10, IgA, and IgM levels in the SYN group, but increased (*P* < 0.05) the plasma IL-1β level in the PRO group compared with the offspring pigs in the maternal supplemented groups. Among the maternal supplemented groups, the PRO group had a lower (*P* < 0.05) plasma IL-1β level in relation to the other three groups, while plasma IL-10 and IgA levels in the PRO group were also lower (*P* < 0.05) compared with those in the CON and SYN groups. In addition, maternal synbiotics supplementation increased (*P* < 0.05) the plasma IgM level in comparison to the CON and PRO groups. Among the maternal-offspring supplemented groups, the SYN group had lower (*P* < 0.05) plasma IL-10, TNF-α, and IgA levels than the CON and PRO groups.

Among the maternal supplemented groups, the ANT and SYN groups had lower (*P* < 0.05) plasma IL-10 and IgA levels compared with the CON and PRO groups, whereas the SYN group had a lower (*P* < 0.05) plasma TNF-α level compared with the CON and PRO groups. In the maternal-offspring supplemented groups, plasma IL-10 level in the ANT group, TNF-α level in the SYN group, and IgA level in the PRO group were lower (*P* < 0.05) compared with the CON group.

### Effects of maternal-offspring supplementation of probiotics and synbiotics on splenic immune response of offspring piglets at 95 and 125 day-old

**Figure 2:**
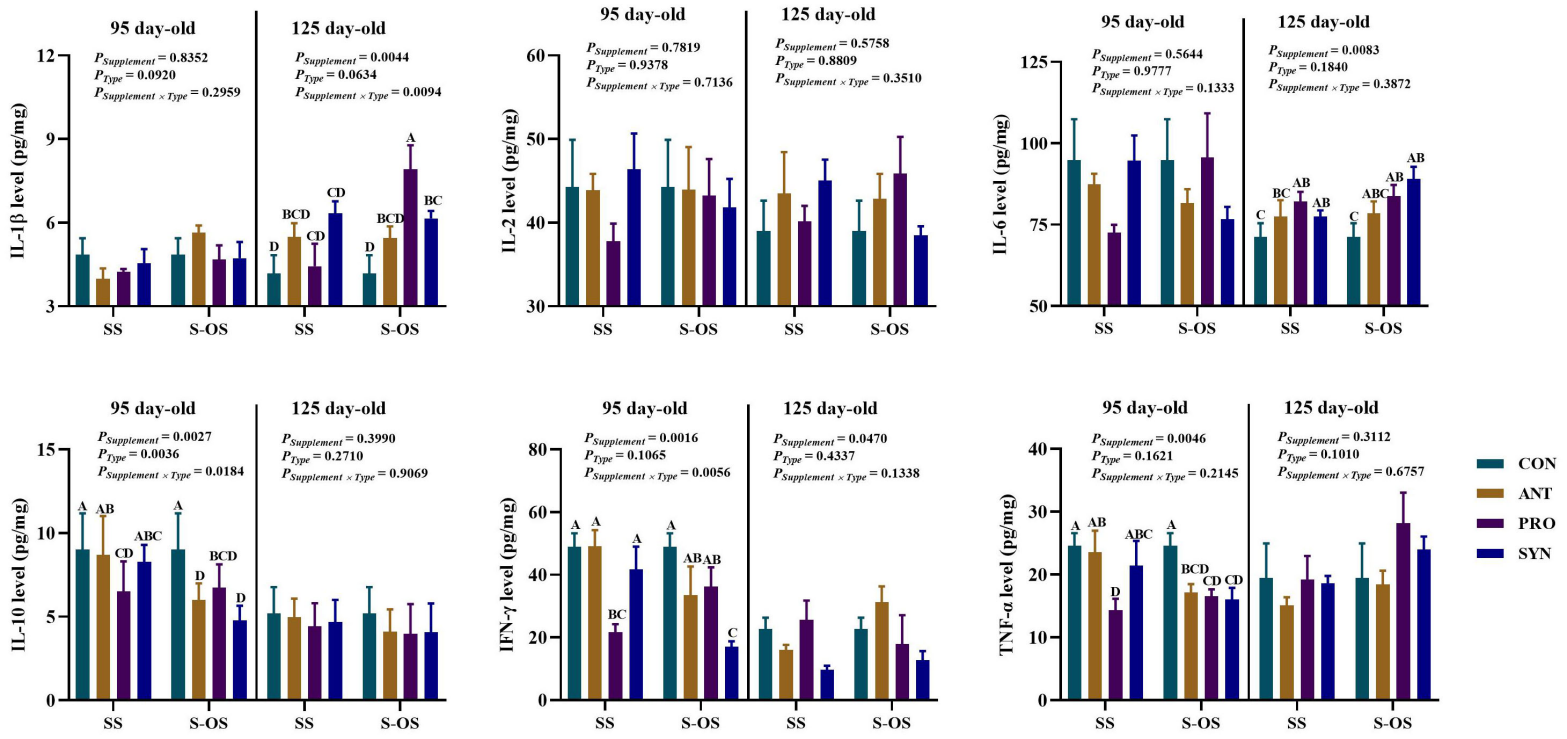
Effects of maternal-offspring supplementation of probiotics and synbiotics on splenic immunocytokines of offspring piglets at 95 and 125 day-old. Data are expressed as means with their pooled SEM (*n* = 6). Different letters (A−D) indicate significant differences among groups (*P* < 0.05). *P*_Supplement_: *P*-values of different kinds of additives in the diet, including no or added antibiotics, probiotics, or prebiotics; *P*_Type_: *P*-values of different ways of supplement, including maternal supplementation and maternal-offspring supplementation; *P*_Supplement_ × *P*_Type_: *P*-values of different kinds of additives and different ways of supplement. CON, control group; ANT, antibiotic group; PRO, probiotics group; SYN, synbiotics group; SS, maternal supplementation; S-OS, maternal-offspring supplementation; IL, interleukin; IFN, interferon; TNF, tumor necrosis factor.

The immunocytokines level in the spleen of offspring pigs at 95 and 125 day-old is shown in **Figure 2**. At 95 day-old, maternal-offspring supplementation decreased (*P* < 0.05) the IL-10 level in the ANT group and the IFN-γ level in the SYN group compared with the maternal supplemented groups. Among the maternal supplemented groups, the PRO group had a lower (*P* < 0.05) IL-10 level than that in the CON and ANT groups, while IFN-γ and TNF-α levels were lower (*P* < 0.05) compared with the other three groups. Among the maternal-offspring supplemented groups, IL-10 and TNF-α levels in the CON group were higher, and IFN-γ level in the SYN group was lower, when compared with the other three groups (*P* < 0.05).

At 125 day-old, maternal-offspring probiotics supplementation increased (*P* < 0.05) the IL-1β level in the spleen of offspring pigs compared with the maternal-supplemented PRO group. Among the maternal supplemented groups, the PRO and SYN groups had a higher (*P* < 0.05) IL-6 level in contrast to the CON group.

**Figure 3:**
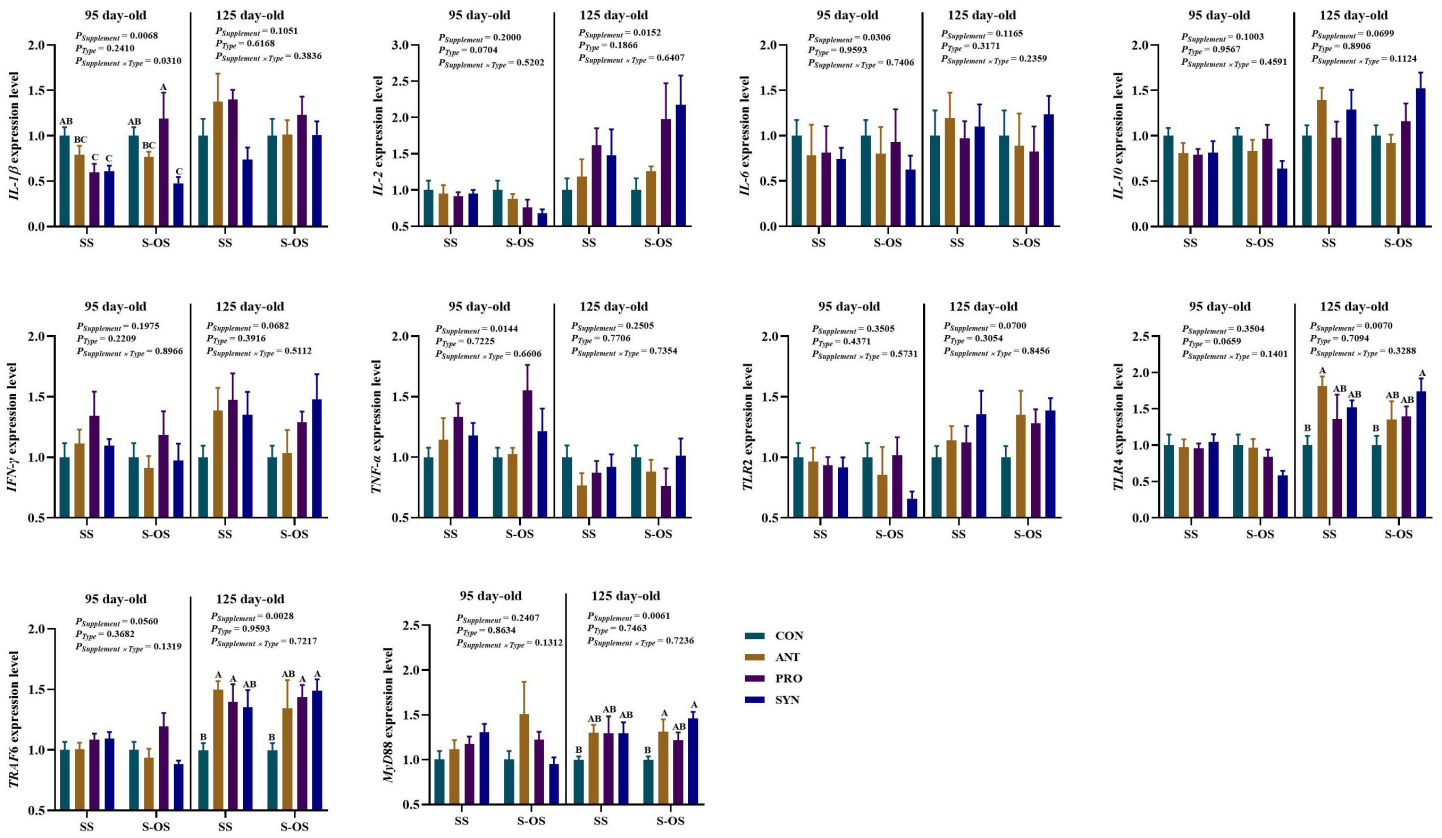
Expression of splenic immune-related genes of offspring piglets at 95 and 125 day-old. Data are expressed as means with their pooled SEM (*n* = 6). Different letters (A−C) indicate significant differences among groups (*P* < 0.05). *P*_Supplement_: *P*-values of different kinds of additives in the diet, including no or added antibiotics, probiotics, or prebiotics; *P*_Type_: *P*-values of different ways of supplement, including maternal supplementation and maternal-offspring supplementation; *P*_Supplement_ × *P*_Type_: *P*-values of different kinds of additives and different ways of supplement. CON, control group; ANT, antibiotic group; PRO, probiotics group; SYN, synbiotics group; SS, maternal supplementation; S-OS, maternal-offspring supplementation; IL, interleukin; TNF, tumor necrosis factor; TLR, toll-like receptors; TRAF6, tumor necrosis factor receptor-associated factor 6; MyD88, myeloid differentiation factor 88; NF-κB, nuclear factor kappa B.

The immune-related gene expressions in the spleen of offspring pigs at 95 and 125 day-old are shown in **Figure 3**. At 95 day-old, maternal-offspring probiotics supplementation upregulated (*P* < 0.05) the *IL-1β* expression compared with the maternal-supplemented PRO group. Among the maternal supplemented groups, the *IL-1β* expression was downregulated (*P* < 0.05) in the PRO and SYN groups compared with the CON group.

At 125 day-old, maternal antibiotics and probiotics supplementation upregulated (*P* < 0.05) the tumor necrosis factor receptor-associated factor 6 (*TRAF6*) expression compared with the maternal-supplemented CON group. Among the maternal-offspring supplemented groups, the *TLR*4 expression was upregulated (*P* < 0.05) in the SYN group in comparison to the CON group. Moreover, the *MyD*88 expression was upregulated (*P* < 0.05) in the ANT group compared with the CON group. However, there were no significant differences (*P* > 0.05) in immune-related gene expressions in the spleen of offspring pigs between maternal and maternal-offspring supplemented groups at 125 day-old.

### Effects of maternal-offspring supplementation of probiotics and synbiotics on ileal immune response of offspring piglets at 95 and 125 day-old

**Figure 4:**
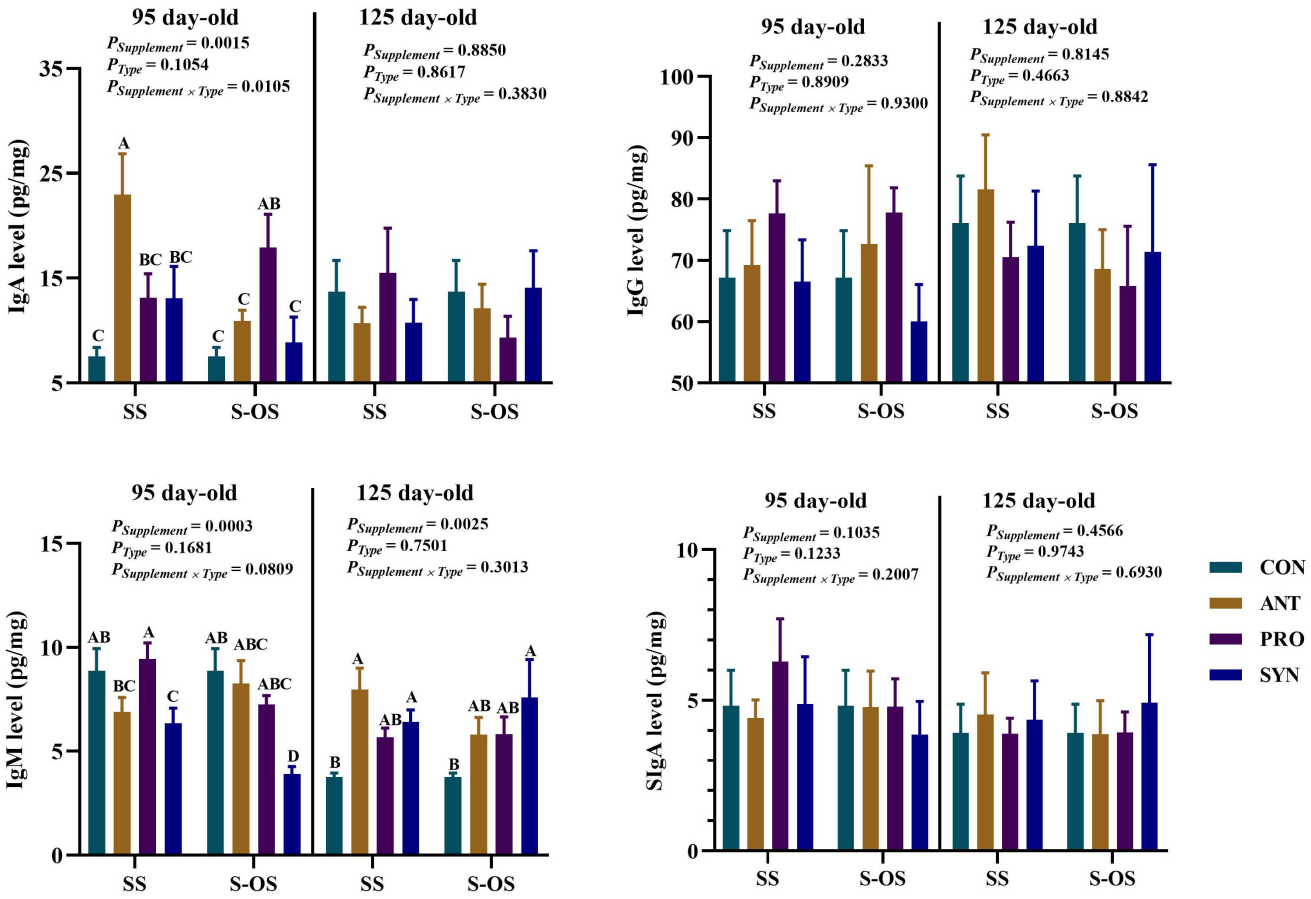
Effects of maternal-offspring supplementation of probiotics and synbiotics on ileal immunoglobulins of offspring piglets at 95 and 125 day-old. Data are expressed as means with their pooled SEM (*n* = 6). Different letters (A−D) indicate significant differences among groups (*P* < 0.05). *P*_Supplement_: *P*-values of different kinds of additives in the diet, including no or added antibiotics, probiotics, or prebiotics; *P*_Type_: *P*-values of different ways of supplement, including maternal supplementation and maternal-offspring supplementation; *P*_Supplement_ × *P*_Type_: *P*-values of different kinds of additives and different ways of supplement. CON, control group; ANT, antibiotic group; PRO, probiotics group; SYN, synbiotics group; SS, maternal supplementation; S-OS, maternal-offspring supplementation; Ig, immunoglobulin; sIgA, Secretory immunoglobulin A.

The effects of maternal-offspring supplementation of probiotics and synbiotics on ileal immunoglobulins of offspring pigs at 95 and 125 day-old are shown in **Figure 4**. At 95 day-old, maternal-offspring supplementation decreased (*P* < 0.05) the IgA level in the ANT group and IgM level in the SYN group compared to the maternal-supplemented groups. Among maternal supplementation groups, the ANT group had a higher (*P* < 0.05) IgA level compared with the other three groups, the SYN group had a lower (*P* < 0.05) IgM level than the CON and PRO groups, and the PRO group had a higher (*P* < 0.05) IgM level than the ANT group.

At 125 day-old, maternal supplementation of antibiotics and synbiotics increased (*P* < 0.05) the IgM level of offspring pigs compared with the maternal-supplemented CON group. Moreover, maternal-offspring supplementation of synbiotics increased (*P* < 0.05) the IgM level of offspring pigs compared with the maternal-offspring CON group.

**Figure 5:**
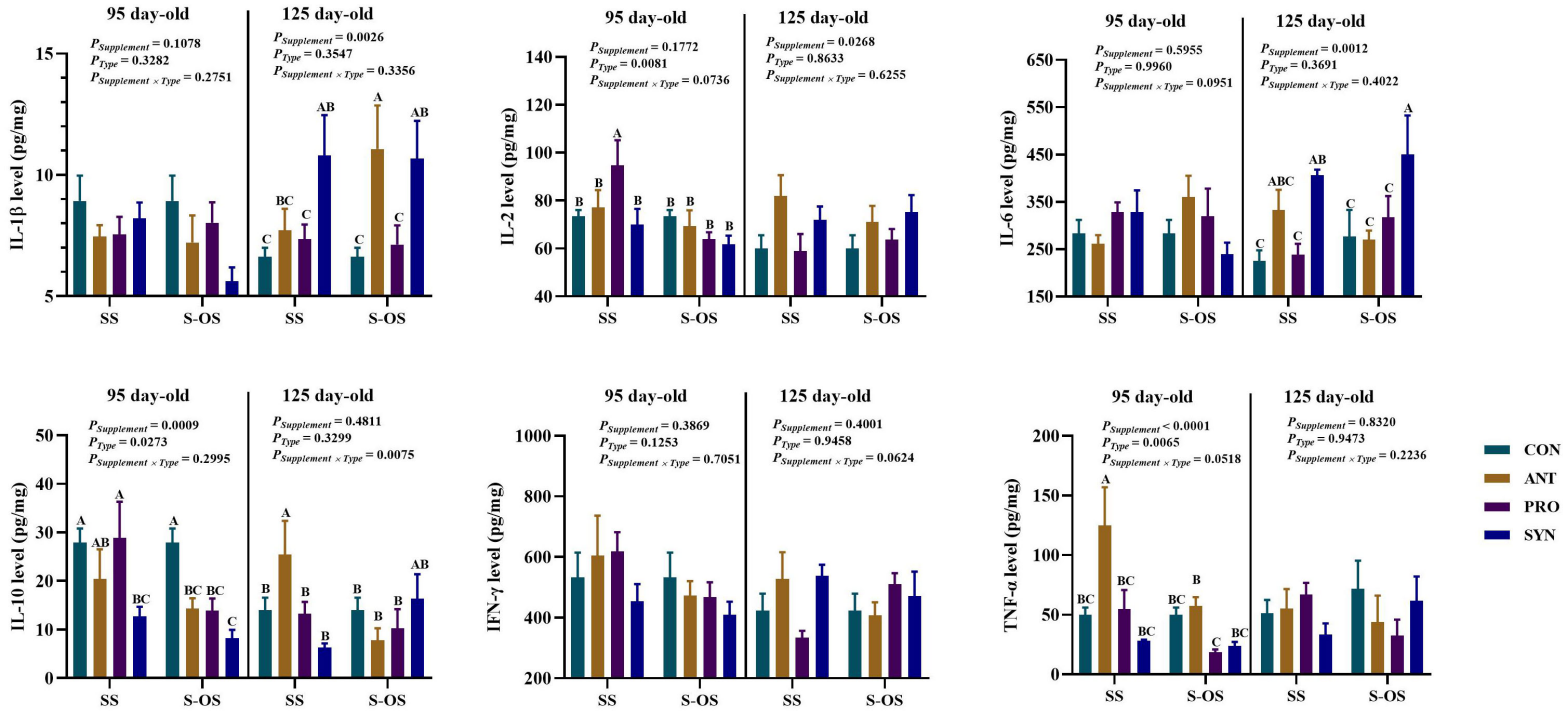
Effects of maternal-offspring supplementation of probiotics and synbiotics on ileal immunocytokines of offspring piglets at 95 and 125 day-old. Data are expressed as means with their pooled SEM (*n* = 6). Different letters (A−C) indicate significant differences among groups (*P* < 0.05). *P*_Supplement_: *P*-values of different kinds of additives in the diet, including no or added antibiotics, probiotics, or prebiotics; *P*_Type_: *P*-values of different ways of supplement, including maternal supplementation and maternal-offspring supplementation; *P*_Supplement_ × *P*_Type_: *P*-values of different kinds of additives and different ways of supplement. CON, control group; ANT, antibiotic group; PRO, probiotics group; SYN, synbiotics group; SS, maternal supplementation; S-OS, maternal-offspring supplementation; IL, interleukin; IFN, interferon; TNF, tumor necrosis factor.

The immunocytokines level in the ileum of offspring pigs at 95 and 125 day-old is shown in **Figure 5**. At 95 day-old, maternal-offspring probiotics supplementation decreased (*P* < 0.05) the IL-2, IL-10, and TNF-α levels compared with the maternal supplementation of probiotics. Among maternal supplementation groups, the PRO group had a higher (*P* < 0.05) IL-2 level compared with the other three groups, as well as the IL-10 level compared with the SYN group, while the ANT group had a higher (*P* < 0.05) TNF-α level compared with the other three groups.

Among the maternal supplementation groups, the SYN group had higher (*P* < 0.05) IL-1β and IL-6 levels compared with the CON and PRO groups, and the ANT group had a higher (*P* < 0.05) IL-10 level than the other three groups. Among the maternal-offspring supplementation groups, the ANT group had a higher (*P* < 0.05) IL-1β level than the CON and PRO groups, and the SYN group had a higher (*P* < 0.05) IL-6 level compared with the other three groups.

**Figure 6:**
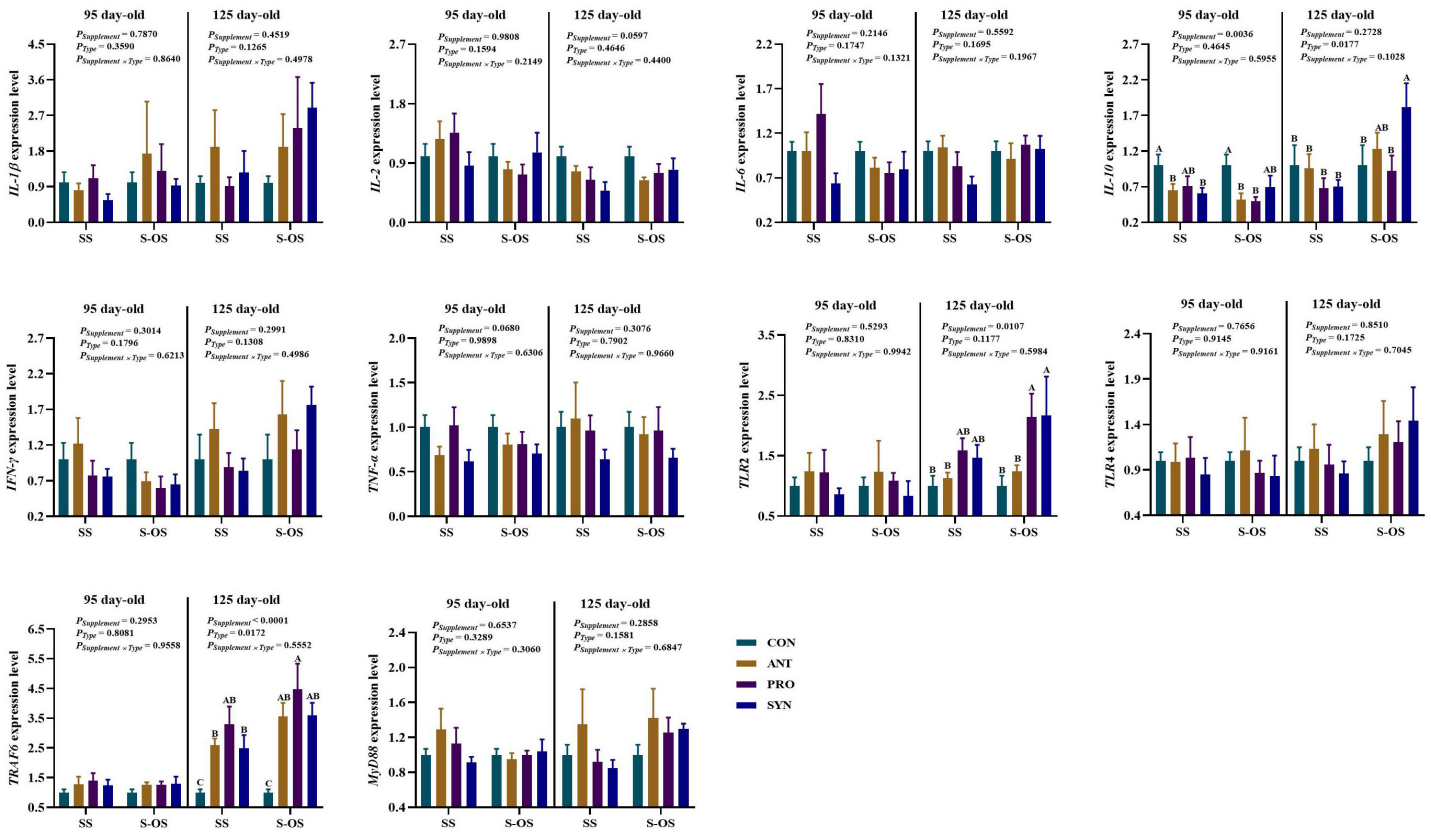
Expression of ileal immune-related genes of pig at 95 and 125 day-old. Data are expressed as means with their pooled SEM (*n* = 6). Different letters (A−C) indicate significant differences among groups (*P* < 0.05). *P*_Supplement_: *P*-values of different kinds of additives in the diet, including no or added antibiotics, probiotics, or prebiotics; *P*_Type_: *P*-values of different ways of supplement, including maternal supplementation and maternal-offspring supplementation; *P*_Supplement_ × *P*_Type_: *P*-values of different kinds of additives and different ways of supplement. CON, control group; ANT, antibiotic group; PRO, probiotics group; SYN, synbiotics group; SS, maternal supplementation; S-OS, maternal-offspring supplementation; *IL*, interleukin; *TNF*, tumor necrosis factor; *TLR*, toll-like receptors; *TRAF6*, tumor necrosis factor receptor-associated factor 6; *MyD88*, myeloid differentiation factor 88; *NF-κB*, nuclear factor kappa B.

The immune-related gene expressions in the ileum of offspring pigs at 95 and 125 day-old are shown in **Figure 6**. At 95 day-old, maternal antibiotics and synbiotics supplementation downregulated (*P* < 0.05) the *IL*-10 expression compared with the maternal CON group. Maternal-offspring antibiotics and probiotics supplementation downregulated (*P* < 0.05) the *IL*-10 expression compared with the maternal-offspring CON group.

At 125 day-old, maternal-offspring synbiotics supplementation upregulated (*P* < 0.05) the *IL*-10 expression compared with the maternal synbiotics supplementation. Maternal antibiotics, probiotics, and synbiotics supplementation upregulated (*P* < 0.05) the *TRAF*6 expression compared with the CON group. Among the maternal-offspring supplementation groups, the *IL*-10 expression was upregulated (*P* < 0.05) in the SYN group compared with the CON and PRO groups, as well as the *TLR*2 expression in the PRO and SYN groups compared with the CON and ANT groups. Moreover, maternal-offspring antibiotics, probiotics, and synbiotics supplementation upregulated (*P* < 0.05) the *TRAF*6 expression compared with the CON group.

## Discussion

Maternal nutrition during pregnancy and lactation plays crucial roles in the overall health status and the postnatal growth and development of offspring, in turn, to the development of the pig industry. Probiotics, as a potential feed additive for swine nutrition, can enhance immunity and reduce the incidence of diarrhea in piglets at early stages of age (17). Moreover, maternal probiotics and synbiotics supplementation could also play an important role in the immune development of offspring (12, 18). Therefore, this study evaluated the effects of maternal and maternal-offspring supplementation of probiotics and synbiotics on the immune function of offspring piglets. The results indicated that different supplemental approaches have differences in the development of immune function in offspring piglets at different stages of age.

Passive immunity can be derived from sows to suckling piglets, as several active antibodies derived from sow colostrum and milk could contribute to the development of active immunity to protect the offspring piglets from different diseases (19). Maternal nutritional strategies during pregnancy and lactation have positive impacts on the immunity of offspring piglets *via* transferring the immune molecules through colostrum (20). In the present study, maternal probiotics supplementation increased the splenic IL-6 level of offspring piglets compared with the CON group at 21 day-old, while maternal/maternal-offspring synbiotics supplementation increased the splenic IL-1β level at 95 day-old and IL-6 and IFN-γ levels at 95 day-old compared with the ANT group. A previous study also reported that adding amoxicillin to the sow diet from the 10^th^ to the 21^st^ day after delivery affected the umami perception and ghrelin regulation in the offspring piglets (21). Research evidence indicated that *Bacillus*-based probiotics supplementation in sow diets increased IL-10 and IgM concentrations in serum and ileum and decreased the TNF-α level in the ileum of offspring piglets, and concluded that the probiotics supplementation protected the intestinal health of piglets by forming biofilm structures in the digestive tract and villi structures (22). Probiotics can improve the host intestinal immune system by enhancing cell-mediated immunity, antibody production, and stimulating T-cell migration (23). Therefore, our findings suggest that maternal antibiotics supplementation adversely affects the immune function, while probiotics supplementation may enhance the anti-inflammatory ability of offspring piglets. Although certain combinations of synbiotics may enhance host immunity synergistically, it is necessary to assess the compatibility of these combinations and their impact on the metabolic molecules of sows and their offspring.

The spleen, as the largest secondary lymphoid organ, is an important site for immune response (24). The physiological position of the spleen allows it to filter pathogens and abnormal cells in the blood and promote low-probability interactions between antigen-presenting cells and homologous lymphocytes (25). Thus, we further evaluated the effects of maternal and maternal-offspring supplementation of probiotics and synbiotics on the splenic immunity of offspring piglets. Our findings showed that maternal-offspring synbiotics supplementation decreased the IL-10 and IFN-γ levels in the spleen of offspring piglets compared with the maternal synbiotics supplementation at 95 day-old, whereas maternal-offspring probiotics supplementation increased the IL-1β level in the spleen of offspring piglets compared with the maternal probiotics supplementation at 125 day-old. IL-10 is a typical anti-inflammatory cytokine and plays an important role in maintaining immune homeostasis (26), whereas IL-1β is a potential pro-inflammatory cytokine that plays a defensive role against host infections and injuries (27). Moreover, a previous study found that IL-10 plays an important regulatory role in piglets infected with porcine circovirus type 2 (28). The research evidence has also shown that MyD88-dependent IL-1β secretion in the spleen leads to neutrophil exosmosis and downregulates *ZO-2* expression to increase endothelial cell permeability (29). Thus, maternal probiotics and synbiotics supplementation presented more beneficial for the immune response of spleen function of offspring piglets at 95 and 125 day-old than that of the maternal-offspring probiotics and synbiotics supplementation.

The small intestine is composed of a single layer of intestinal epithelial cells, which not only plays an important role in the digestion and absorption of nutrients, but also is the largest immune organ of the body, affecting the body’s defense mechanisms (30). Probiotics provide strong protection for the body health by competing with pathogenic bacteria for ecological niches and nutrients and enhancing the intestinal immune system (31, 32). In the present study, maternal-offspring probiotics supplementation decreased the IL-2, IL-10, and TNF-α levels in the ileum of offspring piglets at 95 day-old, but maternal-offspring antibiotics increased the IL-1β level in the ileum at 125 day-old compared with the maternal-supplemented groups. In addition, maternal probiotics and synbiotics supplementation decreased the TNF-α level in the ileum of offspring piglets at 95 day-old compared with the maternal antibiotics supplementation. However, maternal-offspring antibiotics supplementation increased the IL-1β level in the ileum of offspring piglets at 125 day-old compared with the maternal-offspring supplemented CON and PRO groups. Previously, it has been reported that soluble proteins p40 and p75 in the supernatant of *Lactobacillus rhamnosus* GG culture solution could inhibit apoptosis and barrier damage induced by TNF-α in colonic epithelial cells (33). Daily intake of *Lactobacillus plantarum* JL01 (0.5 × 10^10^ CFU) may regulate immunocytokine levels in piglets by downregulating *IL-1β* expression, reducing the accumulation of succinic acid and palmitic acid, and increasing the accumulation of docosahexaenoic acid and tauroursodeoxycholic acid, thereby enhancing immune function in weaned piglets (34). Consistent with the above findings, our findings indicate that both maternal supplementation and the maternal-offspring supplementation of probiotics were more effective in enhancing the ileal immune function of offspring Bama mini-pigs.

## Conclusions

In summary, the immune function of the offspring piglets varied depending on the specific approach used for probiotics and synbiotics supplementation. Compared with the maternal-offspring probiotics and synbiotics supplementation, the maternal supplementation of these feed additives showed more beneficial for spleen function at 95 and 125 day-old. However, maternal-offspring probiotics supplementation was more effective in enhancing ileal immune function than the maternal probiotics supplementation in Bama mini-pigs. These findings provide a physiological and biochemical basis for explaining the stress resistance of Bama mini-pigs and provide technical guidance for the application of probiotics and synbiotics in the immune regulation of “maternal-offspring integration”, which may contribute to the development and utilization of local mini-pig breeds.

## Data Availability

The original contributions presented in the study are included in the article/**Supplementary Material**. Further inquiries can be directed to the corresponding author.
