# Supplementary material for: Effects of maternal-offspring supplementation of probiotics and synbiotics on the immunity of offspring Bama mini-pigs
Source: Front Immunol. 2025 Feb 13;16:1507080. doi: 10.3389/fimmu.2025.1507080 (PMC11864950; doi:10.3389/fimmu.2025.1507080)
Supplement: Supplementary file 1 [file Table1.docx]

**Supplementary Table 1** Primers sequences used for the RT-PCR.

| Gene names | Primer sequence (5′-3′) | Product size (bp) |
| --- | --- | --- |
| *IL-1β* | F: AAGAGGGACATGGAGAAGCGATTTG  R: TTGTTCTGCTTGAGAGGTGCTGATG | 114 |
| *IL-2* | F: AAGCTCTGGAGGGAGTGCTA  R: CAACAGCAGTTACTGTCTCATCA | 115 |
| *IL-6* | F: GCTGCTTCTGGTGATGGCTACTG  R: AGAGGTGAAGAGCATTTTGTCTGAGG | 97 |
| *IL-10* | F: GTCCGACTCAACGAAGAAGG  R: GCCAGGAAGATCAGGCAATA | 106 |
| *IFN-γ* | F: CCATTCAAAGGAGCATGGAT  R: GAGTTCACTGATGGCTTTGC | 146 |
| *TNF-α* | F: ATTCAGGGATGTGTGGCCTG  R: CCAGATGTCCCAGGTTGCAT | 141 |
| *TLR-2* | F: GCAATAATGACACCTTCGCTGAGATTC  R: AGATGGCTGATGTTCTGAATTGACCTC | 139 |
| *TLR-4* | F: AGGACGAAGACTGGGTGAGGAATG  R: CCTGGATGATGTTAGCAGCGATGG | 126 |
| *MyD88* | F: GATGGTAGCGGTTGTCTCTGAT  R: GATGCTGGGGAACTCTTTCTTC | 146 |
| *NF-κB* | F: AGTACCCTGAGGCTATAACTCGC  R: TCCGCAATGGAGGAGAAGTC | 109 |
| *TRAF6* | F: CAAGAGAATACCCAGTCGCACA  R: ATCCGAGACAAAGGGGAAGAA | 114 |
| *β-actin* | F: GATCTGGCACCACACCTTCTACAAC  R: TCATCTTCTCACGGTTGGCTTTGG | 107 |

*IL,* interleukin; *IFN-γ*, interferon-γ; *TNF-α*, tumor necrosis factor-α; *TLR*, toll-like receptor; *MyD88*, myeloid differentiation factor 88; *NF-κB*, nuclear factor kappa B; *TRAF6*, tumor necrosis factor receptor-associated factor 6.
